# No association between *TP53* Arg72Pro polymorphism and ovarian cancer risk: evidence from 10113 subjects

**DOI:** 10.18632/oncotarget.22603

**Published:** 2017-11-21

**Authors:** Anqi Zhang, Ting-Yan Shi, Yuan Zhao, Junmiao Xiang, Danyang Yu, Zongwen Liang, Chaoyi Xu, Qiong Zhang, Yue Hu, Danhan Wang, Jing He, Ping Duan

**Affiliations:** ^1^ Department of Obstetrics and Gynecology, The Second Affiliated Hospital and Yuying Children's Hospital of Wenzhou Medical University, Wenzhou 325027, Zhejiang, China; ^2^ Department of Obstetrics and Gynecology, Zhongshan Hospital, Fudan University, Shanghai 200032, China; ^3^ Department of Pediatric Surgery, Guangzhou Institute of Pediatrics, Guangzhou Women and Children's Medical Center, Guangzhou Medical University, Guangzhou 510623, Guangdong, China

**Keywords:** TP53, polymorphism, ovarian cancer, susceptibility, meta-analysis

## Abstract

The *TP53* gene product is an important regulator of cell growth and a tumor suppressor. The association between *TP53* Arg72Pro polymorphism and ovarian cancer risk has been widely investigated, but the results are contradictory. We therefore searched the PubMed, EMBASE and Chinese Biomedical databases for studies on the relation between *TP53* Arg72Pro polymorphism and ovarian cancer risk. Our final meta-analysis included 24 published studies with 3271 cases and 6842 controls. Pooled results indicated that there was no significant association between *TP53* Arg72Pro polymorphism and ovarian cancer risk [Pro/Pro vs. Arg/Arg: odds ratio (OR) =1.04, 95% confidence interval (CI) = 0.81-1.34; Arg/Pro vs. Arg/Arg: OR = 1.14, 95% CI = 0.96-1.36; recessive: OR = 1.05, 95% CI = 0.90-1.22; dominant: OR = 1.12, 95% CI = 0.94-1.33; and Pro vs. Arg: OR = 1.06, 95% CI=0.93-1.20]. Likewise, stratified analyses failed to reveal a genetic association. Despite some limitations, the present meta-analysis provides statistical evidence indicating a lack of association between *TP53* Arg72Pro polymorphism and ovarian cancer risk.

## INTRODUCTION

With 238,700 new cases worldwide, ovarian cancer was the seventh most frequently occurring cancer among women in 2012, and was responsible for 151,900 deaths. In developing countries, newly diagnosed cases of ovarian cancer have exceeded those of cervical cancer [1]. Moreover, mortality from ovarian cancer has decreased by only 1.29 per 100,000 over the past several decades due in large part to the paucity of effective screening methods and chemopreventive agents, and to its being asymptomatic during early stages [2, 3]. Although active surgical treatment and adjuvant chemotherapy have been applied to the treatment of advanced ovarian cancer, the prognosis of the patients remains poor with a 5-year survival rate of only 23% [4, 5]. Considerable research has focused on explaining the molecular mechanisms underlying ovarian cancer, but in the absence of an appropriate progression model, they remain far from clear [6, 7]. Thus, identification of a sensitive and early-detected biomarker useful for cancer prediction and prevention is badly needed.

*TP53* is a tumor suppressor gene is located on the chromosome 17p13 short arm and encodes a protein with 393 amino acids [8]. Its product, p53, is regarded as a major inhibitor of tumorigenesis, which is involved in cycle arrest, DNA repair, apoptosis or cellular aging [9, 10]. Mutations in *TP53* can result in Li–Fraumeni syndrome, which increases the risk of diverse cancers, including breast cancer, carcinosarcoma, leukemia and brain tumors, among others [11]. In addition to the tumor-associated *TP53* mutations, polymorphisms also have an important impact on the susceptibility to cancer [12–15]. The well-studied *TP53* polymorphism is at codon 72 of exon 4 (CGC to CCC) and corresponds to a change from arginine to proline [16, 17]. The two polymorphic forms of *TP53* exhibit differences in their biological function. Whereas the Arg72 form induces cell apoptosis upon stress and inhibits tumorigenesis, the Pro72 inhibits the G1 phase of cell cycle progression [18]. There has been much research on the relationship between *TP53* codon 72 polymorphism and ovarian cancer risk, but the results are conflicting and inconsistent. Some studies have shown that the *TP53* Arg allele is associated with a higher risk of ovarian cancers [19–21], while others found that the *TP53* Pro allele is likely a risk factor for ovarian cancer [22–24]. In addition, there are also publications suggesting the *TP53* Arg72Pro polymorphism is not associated with susceptibility to ovarian cancer [25, 26]. The reasons for the contradictory results may be attributable to inadequate sample size, different sources of DNA, ethnicity, different environmental exposures, or the borderline effect of genetic variant. In an effort to increase clarity, we collected all eligible studies and analyzed the potential effect of TP53 codon 72 polymorphism on the susceptibility to ovarian cancer.

## RESULTS

### Study characteristics

**Figure 1:**
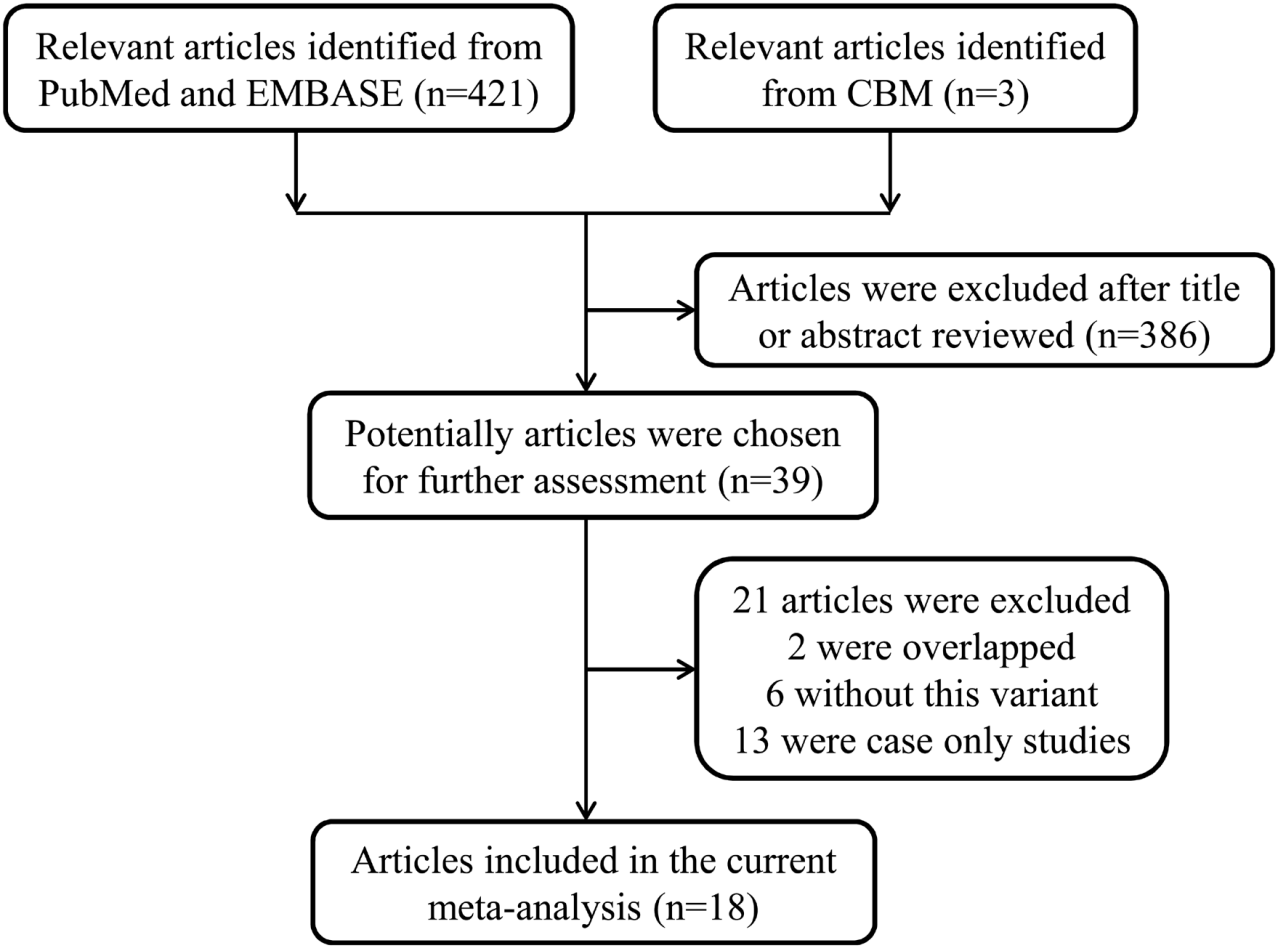
Flowchart of included studies

Through searches of the PubMed, EMBASE and Chinese Biomedical (CBM) databases, a total of 424 potentially relevant articles were initially identified. Of those, 386 were excluded, and 38 were chosen for further evaluation through checking of the title and abstract (Figure 1). Based on the inclusion criteria described below in the Methods, 18 articles were included in the final analysis [19, 20, 22–36]. Among those 18, the publication by Schildkraut et al. [26] investigated the genotype distributions of the *TP53* Arg72Pro polymorphism in different areas, so it was divided into seven separate studies. Overall, 24 case-control studies with 3271 cases (13 to 626 per study) and 6842 controls (13 to 1045 per study) were included in the present meta-analysis. The main characteristics of all the included studies are presented in Table 1. Among these studies, 19 were conducted with Caucasians, four with Asians, and only one with Africans. Because detailed information about the source of the controls were not been provided in the study by Peller et al. [36], only 12 articles were classified as population-based, and 11 were hospital-based. In addition, 15 studies with a quality score ≥ 9 were considered to be high quality, while nine with a score < 9 were regarded as low quality. Table 1 also summarizes the distribution of genotypes, minor allele frequencies (MAFs) and the Hardy–Weinberg equilibrium (HWE) in the controls.

**Table 1:** Characteristics of studies included in the current meta-analysis

Surname	Year	Country	Ethnicity	Design	Genotyping method	Case				Control				MAF	HWE	Score
						AA	AP	PP	All	AA	AP	PP	All			
Buller	1997	USA	Caucasian	PB	PCR-SSCP	98	79	13	190	30	18	4	52	0.25	0.579	10
Peller	1999	Israel	Caucasian	Not detailed	DS	7	6	0	13	8	5	0	13	0.19	0.447	5
Hogdall	2002	Denmark	Caucasian	PB	PCR-RFLP	118	73	20	211	48	27	8	83	0.26	0.165	9
Li	2002	China	Asian	PB	PCR-RFLP	14	20	5	39	29	67	35	131	0.52	0.920	10
Qie	2002	China	Asian	HB	PCR-RFLP	10	18	2	30	12	16	2	30	0.33	0.273	5
Pegoraro	2003	South Africa	African	HB	AS-PCR	9	29	25	63	32	147	161	340	0.69	0.852	6
Agorastos	2004	Greece	Caucasian	HB	PCR	26	22	3	51	6	19	5	30	0.48	0.142	5
Kang	2004	China	Asian	HB	PCR	28	60	36	124	37	64	27	128	0.46	0.945	9
Morari	2006	Brazil	Caucasian	PB	AS-PCR	23	46	0	69	117	91	14	222	0.27	0.505	9
Santos	2006	Portugal	Caucasian	HB	AS-PCR	49	40	10	99	117	58	13	188	0.22	0.128	7
Ueda	2006	Japan	Asian	HB	PCR-RFLP	21	41	6	68	34	54	7	95	0.36	0.021	6
Schildkraut-POCS	2009	Poland	Caucasian	PB	TaqMan	51	63	4	118	368	207	45	620	0.24	0.038	11
Schildkraut-NCOCS	2009	USA	Caucasian	PB	IGGA	132	104	16	252	231	182	24	437	0.26	0.122	13
Schildkraut-MAYO	2009	USA	Caucasian	PB	IGGA	96	82	14	192	261	157	37	455	0.25	0.057	13
Schildkraut-AUS	2009	Australia	Caucasian	PB	MassARRAY	121	59	14	194	219	110	31	360	0.24	0.002	11
Schildkraut-HAW	2009	USA	Caucasian	PB	TaqMan	18	12	0	30	78	60	8	146	0.26	0.416	12
Schildkraut-MAL	2009	Denmark	Caucasian	PB	TaqMan	134	94	25	253	564	371	78	1013	0.26	0.123	14
Schildkraut-SEA	2009	New-England	Caucasian	PB	TaqMan	119	75	18	212	461	326	55	842	0.26	0.796	14
Matei	2012	Roman	Caucasian	HB	PCR-RFLP	9	6	6	21	7	7	7	21	0.50	0.127	4
Dholariya	2013	North India	Caucasian	HB	ASO-PCR	33	50	17	100	62	32	6	100	0.22	0.499	9
Malisic	2013	Serbia	Caucasian	HB	PCR-RFLP	22	22	3	47	45	22	3	70	0.20	0.881	6
Medrek	2013	Poland	Caucasian	PB	TaqMan	302	265	59	626	537	436	72	1045	0.28	0.191	12
Tecza	2015	Poland	Caucasian	HB	PCR-RFLP	130	79	16	225	167	150	24	341	0.29	0.213	11
Benhessou	2016	Morocco	Caucasian	HB	AS-PCR	33	10	1	44	43	27	10	80	0.29	0.095	5

AA, Arg/Arg; AP, Arg/Pro; PP, Pro/Pro; MAF, minor allele frequency; HWE, Hardy-Weinberg equilibrium; PB, population based; HB, hospital based; PCR, polymerase chain reaction; PCR-RFLP, PCR-restriction fragment length polymorphism; AS-PCR, allele-specific PCR; ASO-PCR, allele specific oligonucleotide; IGGA, Illumina golden gate assay; DS, direct sequencing; PCR-SSCP, PCR-single-strand conformation polymorphism assay.

### Meta-analysis

**Figure 2:**
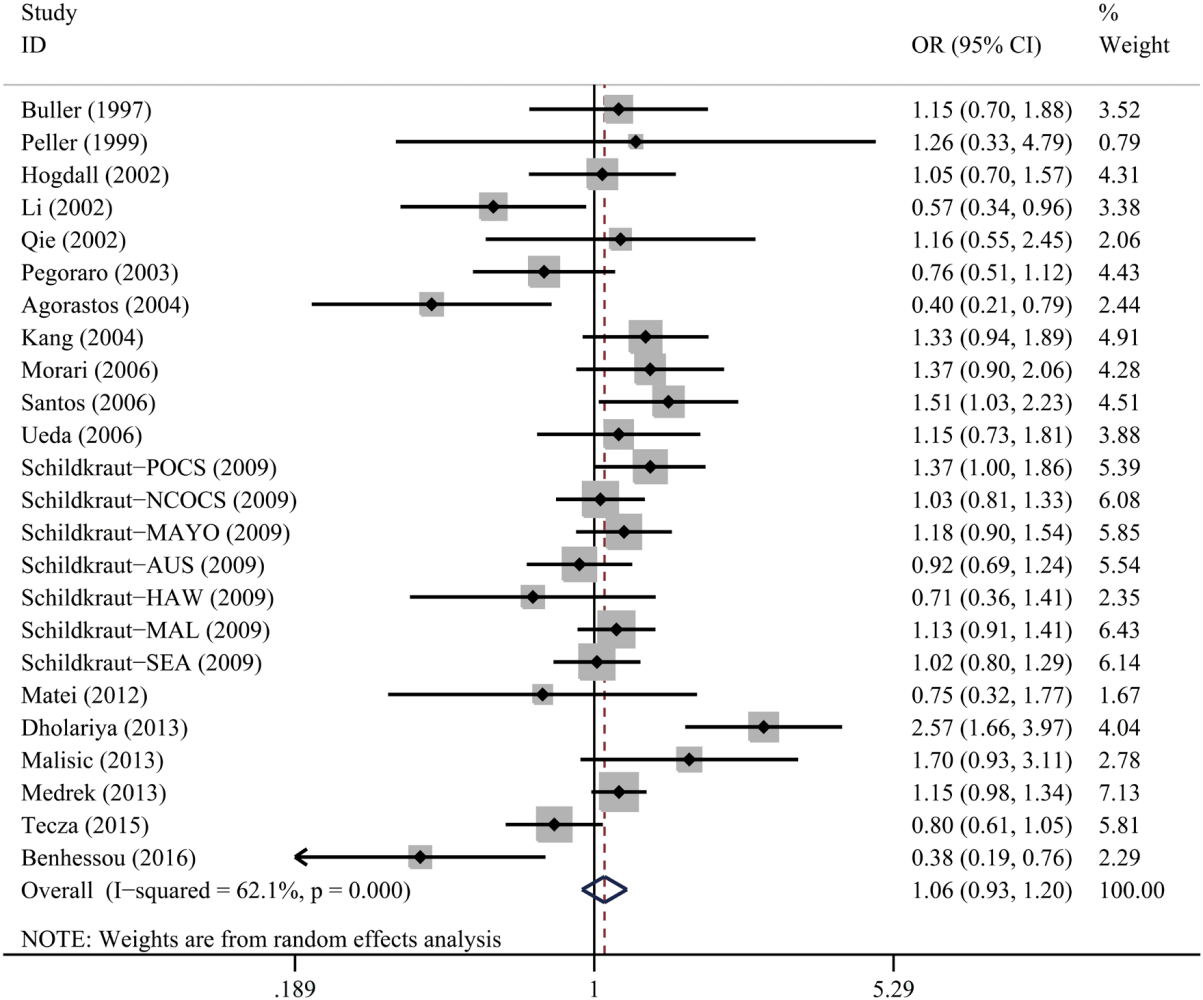
Forest plot for *TP53* Arg72Pro polymorphism and ovarian cancer risk by allele comparison model For each study, the estimation of OR and 95% CI were plotted with a box and a horizontal line. The symbol filled diamond indicates pooled OR and 95% CI.

As shown in Table 2 and Figure 2, the overall pooled analysis indicated no significant association between *TP53* Arg72Pro polymorphism and ovarian cancer risk in any of the five genetic models [homozygous: odds ratio (OR)=1.04, 95% confidence interval (CI)=0.81-1.34; heterozygous: OR=1.14, 95% CI=0.96-1.36; recessive: OR=1.05, 95% CI=0.90-1.22; dominant: OR=1.12, 95% CI=0.94-1.33 and allele comparing: OR=1.06, 95% CI=0.93-1.20). In addition, when we performed subgroup analyses based on ethnicity, source of controls, and quality of studies, there were again no significant results indicating a relationship between the *TP53* Arg72Pro polymorphism and ovarian cancer risk.

**Table 2:** Meta-analysis of the association between *TP53* codon 72 (rs1042522 G>C) polymorphism and ovarian cancer risk

Variables	No. ofstudies	Samplesize	Homozygous		Heterozygous		Recessive		Dominant		Allele	
			OR (95% CI)	*P* ^het^	OR (95% CI)	*P* ^het^	OR (95% CI)	*P* ^het^	OR (95% CI)	*P* ^het^	OR (95% CI)	*P* ^het^
All	24	3271/6842	1.04 (0.81-1.34)	0.015	1.14 (0.96-1.36)	<0.001	1.05 (0.90-1.22)	0.131	1.12 (0.94-1.33)	<0.001	1.06 (0.93-1.20)	<0.001
Ethnicity												
Caucasians	19	2947/6118	1.09 (0.84-1.43)	0.035	1.18 (0.97-1.43)	<0.001	1.09 (0.91-1.29)	0.177	1.15 (0.95-1.39)	<0.001	1.09 (0.95-1.25)	<0.001
Asians	4	261/384	0.99 (0.40-2.42)	0.070	1.08 (0.75-1.56)	0.508	1.07 (0.70-1.65)	0.166	1.06 (0.67-1.68)	0.190	1.02 (0.69-1.49)	0.062
Africans	1	63/340	0.55 (0.24-1.29)	—	0.70 (0.30-1.63)	—	0.73 (0.42-1.27)	—	0.62 (0.28-1.38)	—	0.76 (0.51-1.12)	—
Source of control												
PB	12	2386/5406	1.08 (0.86-1.36)	0.306	1.18 (0.98-1.41)	0.006	1.03 (0.86-1.25)	0.200	1.15 (0.98-1.34)	0.030	1.09 (0.99-1.19)	0.262
HB	11	872/1423	1.06 (0.61-1.84)	0.004	1.05 (0.71-1.55)	<0.001	1.08 (0.83-1.40)	0.135	1.03 (0.67-1.57)	<0.001	1.02 (0.75-1.39)	<0.001
Quality score												
≥9	15	2835/5975	1.13 (0.88-1.47)	0.054	1.19 (0.98-1.44)	<0.001	1.11 (0. 94-1. 32)	0.124	1.17 (0.98-1.41)	<0.001	1.11 (0.98-1.25)	0.002
<9	9	436/867	0.77 (0.39-1.49)	0.064	0.98 (0.63-1.50)	0.033	0.81 (0.56-1.16)	0.425	0.91 (0.56-1.47)	0.004	0.91 (0.64-1.29)	0.002

Het, heterogeneity; OR, odds ratio; CI, confidence interval; HB, hospital based; PB, population based.

### Heterogeneity and sensitivity analyses

As shown in Table 2, significant heterogeneities were observed among studies in the homozygous (*P* = 0.015), heterozygous (*P* < 0.001), recessive (*P* = 0.131) and dominant (*P* < 0.001) models, as well as with allele comparing (*P* < 0.001). We adopted the random-effects model to solve the significant heterogeneity between studies, because it generated wider CIs to estimate genetic susceptibility. To confirm the impact of each study on summary ORs, sensitivity analysis was conducted by separately omitting each single study involved in the analysis and recalculating ORs and the 95% CIs. This analysis showed our results to be statistically robust, as the corresponding pooled ORs and 95% CIs were not materially changed by any of the omissions (data not shown).

### Publication bias

**Figure 3:**
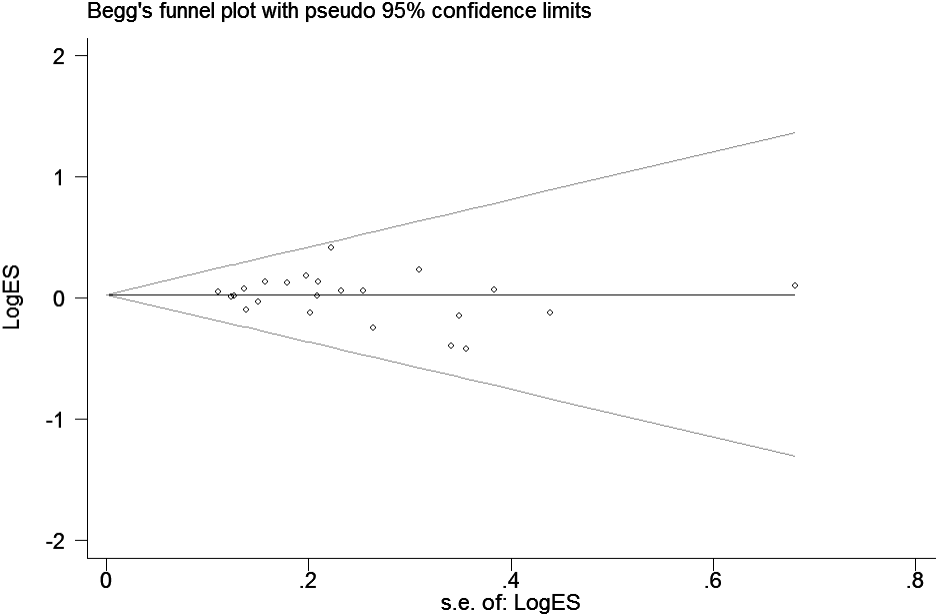
Funnel plot analysis to detect publication bias for *TP53* Arg72Pro polymorphism under allele model Each point represents a separate study for the indicated association.

In this meta-analysis, publication bias was assessed using Begg's and Egger's tests (Figure 3). The results demonstrated that there was no potential publication bias present in the included articles under three genetic models (heterozygous: *P* = 0.945; dominant model: *P* = 0.752 and allele comparing model: *P* = 0.371). However, it was important to note that obvious bias existed in the other two models (homozygous: *P* = 0.027 and recessive model: *P* = 0.032). The reason for the bias may be related to the small sample size so that no single study was considered to be the cause of the publication bias.

## DISCUSSION

The tumor-suppressor protein p53 is a well-known deterrent to cell growth, which inhibits tumorigenesis by activating apoptotic machinery [37]. *TP53* Arg72Pro, a common *TP53* polymorphism, induces certain conformational p53 mutants and binds stably to another tumor suppressor, p73 [38, 39]. The two polymorphic variants of *TP53* exert different biochemical and biological effects on cell cycle progression. The Arg72 form induces higher levels of apoptosis than the Pro72 form, while the Pro72 form has the ability to induce growth arrest during the G1 phase of the cell cycle [40]. We therefore hypothesized that *TP53* Arg72Pro polymorphism may be closely related to the risk of ovarian cancer. Although numerous studies have investigated the relationship between the *TP53* Arg72Pro polymorphism and ovarian cancer risk, the results have been inconsistent. Hence, the current meta-analysis of 24 studies was employed further investigate the association between *TP53* Arg72Pro polymorphism and ovarian cancer, which is the most comprehensive analysis to date. The overall pooled results suggest there is no significant connection between the *TP53* Arg72Pro polymorphism and ovarian cancer risk in five genetic models. This result was further confirmed in analyses stratified based on ethnicity, source of control and quality score. Consistent with the results of present meta-analysis, some earlier studies found that there is little or no association between *TP53* Arg72Pro polymorphism and ovarian cancer risk. Likewise, an previous meta-analysis conducted by Francisco et al. [41] detected no association between the *TP53* Arg72Pro polymorphism and ovarian cancer risk under any genetic model, and stratified analyses also failed to validate a genetic association. On the other hand, a study by Shen et al. [42] concluded that the *TP53* Arg72 allele was associated with a modest, but significantly reduced risk of ovarian cancer when the included studies were classified as high quality. In yet another analysis, the main findings of 19 case-control studies, including 2,240 cases and 5,246 controls, were similar to those of the present study, though the subgroup analysis indicated a marginal association between the *TP53* Arg72Pro polymorphism and ovarian cancer risk in the heterozygote model in Caucasians [43].

One possible explanation for why it is difficult to detect an effect of *TP53* codon 72 polymorphism on ovarian cancer based on these epidemiological results is that there was loss of heterozygosity (LOH) at the *TP53* locus. Based on the previous publications, the *TP53* Arg allele is preferentially mutated and expressed, while the Pro allele is lost in *TP53* Arg72/Pro72 heterozygotes across several cancer types [44, 45]. Thus the results of these genetic association studies may be biased by preferential LOH of the Pro allele. In addition, the results were probably influenced by individual infections with tumor-associated human papillomaviruses (HPVs). Storey found that the Arg 72 isoform was more susceptible to degradation by E6 protein of HPV16 than Pro 72 isoform in a heterozygous situation [46]. However, the original data on HPV infection were unavailable from the included studies, so further stratified analysis based on HPV infection status was not done.

The merits of the present meta-analysis in the context of the previously published ones are as follows. (a) We used an expanded retrieval range that included the latest studies and minimized selection bias. (b) There was a larger sample size and greater statistical power. (c) Our study is further verification that the *TP53* Arg72Pro polymorphism has no impact on ovarian cancer risk. Nonetheless, there are still several limitations to the present meta-analysis. First, the sample sizes of many of the included studies were small, which contributed to reducing the statistical power of the genetic association estimate. Second, this meta-analysis only included publications in English and Chinese, which could lead to selection bias. Third, there were three studies in which the detected genotype frequencies deviated from the HWE [26, 31], making biased results inevitable. Fourth, significant heterogeneity was present under four genetic models, which may be attributable to ethnic differences and/or variation in sample size, study design or genotyping methods. Fifth, the well-known protective factors for ovarian cancer, such as bearing children, oral contraceptives and breastfeeding, were not taken into account due to a lack of individual information. Finally, we did not consider the possibility of the gene-gene interaction.

Despite of these limitations, the pooled results demonstrated that there was no significant association between the *TP53* Arg72Pro polymorphism and ovarian cancer risk. Future well-designed studies that include large samples and take environmental factors into account will be necessary to validate our findings.

## MATERIALS AND METHODS

### Identification of relevant studies

We conducted a search of the PubMed, EMBASE and Chinese Biomedical (CBM) databases using the following keywords: “*TP53* or *p53*”, “polymorphism or variant or variation” and “ovarian” (prior to May 1, 2017). In addition, the references lists of the original articles and reviews were checked manually to identify additional studies. For overlapping data and republished studies, only the latest and largest studies were included in the current meta-analysis.

### Inclusion and exclusion criteria

Inclusion criteria for studies were as follows: (a) evaluation of the association between the *TP53* gene Arg72Pro polymorphism and ovarian cancer risk, (b) case-control design, (c) sufficient and adequate data provided to calculate crude OR and 95% CI, (d) the data were reported in English or Chinese.

Studies were excluded for the following reasons: (a) duplicate publication, (b) cases only studies, or (c) article was a review, case report, editorial or expert opinion.

### Data extraction

Two reviewers (Anqi Zhang and Jing He) assessed weather the retrieved studies met the inclusion criteria, and extracted data from each eligible study. If the two reviewers did not separately reach a unanimous decision on any one item, the dispute was resolved by joint review and consensus. The information retrieved from each study was as follows: the first author's surname, publication data, country of origin, ethnicity, source of control, total number of cases and controls, genotype methods, and numbers of cases and controls for the *TP53* Arg72Pro. Based on differently stratified analyses, ethnicity was divided into three categories: Asians, Caucasians and Africans, and controls were divided into hospital-based and population-based.

### Statistical methods

The strength of the association between *TP53* Arg72Pro polymorphism and ovarian cancer susceptibility was recalculated using crude ORs with the corresponding 95% CIs. The pooled ORs were calculated for the homozygous (Pro/Pro vs. Arg/Arg), heterozygous (Arg/Pro vs. Arg/Arg), dominant [(Arg/Pro + Pro/Pro) vs. Arg/Arg], and recessive [Pro/Pro vs. (Arg/Pro + Arg/Arg)] models, as well as allele comparison (Pro vs. Arg). The Chi square-based Q test was used to calculate the heterogeneity between studies. If the *P* value was less than 0.1, the pooled ORs were calculated using a random-effects model (the DerSimonian and Laird method) [47]. Otherwise, a fixed-effect model (the Mantel–Haenszel method) was chosen for further calculation [48]. Subgroup analyses were classified into three parts: ethnicity, study design and quality score. The quality score for each study was assessed in accordance with the evaluation criteria, as described previously [49–51]. The quality scores ranged from 0 to 15, with scores ≥ 9 defined as high quality, while < 9 were defined as low quality. Publication bias was assessed by constructing a funnel plots, after which the asymmetry of the funnel plots was assessed using Egger's linear regression test [52]. HWE was evaluated using the chi-square-based Q-test, values of *P* < 0.05 were regarded as deviations from the HWE. All statistical tests were conducted using STATA software (version 11.0; Stata Corporation, College Station, TX). Values of *P* < 0.05 was considered significant, and all tests were two-sided.
